# Correlated evolution of neck length and leg length in birds

**DOI:** 10.1098/rsos.181588

**Published:** 2019-05-08

**Authors:** Christine Böhmer, Olivia Plateau, Raphäel Cornette, Anick Abourachid

**Affiliations:** 1 UMR 7179 CNRS/MNHN, Département Adaptations du Vivant, Muséum National d'Histoire Naturelle, 55 rue Buffon, 75005 Paris, France; 2 UMR 7205 Institut de Systématique, Evolution, Biodiversité (ISYEB), Muséum National d'Histoire Naturelle, CNRS, Sorbonne Université, EPHE, CP 50, 57 rue Cuvier, 75005 Paris, France

**Keywords:** numerical variation, scaling, body size, development, evolution, Aves

## Abstract

Despite a diversity of about 10 000 extant species, the sophisticated avian ‘body plan’ has not much changed once it was achieved around 160 Ma after the origin of powered flight. All birds are bipedal having wings, a rigid trunk, a short and ossified tail, a three-segmented leg and digitigrade feet. The avian neck, however, has always been regarded as a classic example of high variability ranging from short necks in songbirds to extremely long, serpentine necks in herons. Yet, the wide array of small to very large species makes it difficult to evaluate the actual neck length. Here, we investigate the evolution of the vertebral formulae in the neck of birds and the scaling relationships between skeletal dimensions and body size. Cervical count in birds is strongly related to phylogeny, with only some specialists having an exceptional number of vertebrae in the neck. In contrast with mammals, the length of the cervical vertebral column increases as body size increases and, thus, body size does not constrain neck length in birds. Indeed, neck length scales isometrically with total leg length suggesting a correlated evolution between both modules. The strong integration between the cervical and pelvic module in birds is in contrast with the decoupling of the fore- and hindlimb module and may be the result of the loss of a functionally versatile forelimb due to the evolution of powered flight.

## 1. Introduction

The evolution of powered flight and the highly specialized morphology of the flight apparatus constrained the functional versatility of the forelimbs. However, in contrast with bat wings that have retained many of the functions of other mammalian forelimbs including the ability to grasp and handle food [1,2], the wings in birds are functionally more limited [3]. Even in birds that secondarily lost the ability to fly, such as the flightless emu or penguin, the forelimbs are either vestigial or adapted for other locomotor modes. Consequently, the neck in birds is not a simple connection between the head and the body, but a highly complex structure that performs a variety of demanding tasks, including feeding, manipulation, preening, sexual display, nest building and combat behaviour (e.g. [4–7]). It contributes to the stabilization of vision during terrestrial locomotion (i.e. head bobbing) [8,9]. During aerial locomotion, the neck is also involved in head stabilization without which the animals are not able to maintain controlled flight (e.g. [10,11]). Most impressively, the latter is illustrated by the remarkable whiffling manoeuvre in geese. The bird briefly flies upside down by rolling its body 180° but keeps its head fixed horizontally [12]. Additionally, there are specialist bird species that actively use their neck during arboreal locomotion (tripodal locomotion in parrots) [13] and cavity excavation (pecking in woodpeckers) [14]. Thus, the avian neck can be regarded as the functional equivalent to the arm and in combination with the beak, the head–neck system of birds even acts as a tool-bearing arm.

The structural basis for all these performances is the cervical musculoskeletal system. In particular, vertebral morphology and number significantly contribute to the biomechanics of the neck in birds (e.g. [4,5,15]). Strikingly, neck length as well as the number of cervical vertebrae (CV) vary greatly among different bird species, ranging from as few as 10 in parrots to as many as 26 in swans. The actual length of the cervical vertebral column is often hidden by the plumage and the neck may be more or less retracted which influences its external appearance. Furthermore, the number of CV is not necessarily an indicator of the length of the neck since a few elongated vertebrae (e.g. [16] in flamingos) may form an equally long and mobile neck as do many short vertebrae (e.g. in swans). In summary, these observations raised the question of what is a long neck in birds.

The aim of the present study is to illuminate the evolution of the vertebral formulae in the neck of birds and the scaling relationships between skeletal dimensions and body size by addressing the following questions: (i) What are the constraints and selective forces on the cervical count in birds? Phylogeny, development and function are potentially involved in contributing to the variation in the number of vertebrae in the neck, but to what extent? (ii) How does neck length relate to body size in birds? Altering body size clearly has biomechanical consequence (e.g. [17]), but to date we lack knowledge about the relation to neck length. (iii) How is individual vertebral length related to the length of other skeletal body parts? Interestingly, we observed that the short-legged swan appears to have relatively short CV, whereas the long-legged flamingo appears to have relatively long CV. Here, we will test if this holds true for a broad sample of birds. Eventually, the results will provide new insights into the functional morphology, development and evolution of the neck in birds.

## 2. Material and methods

### 2.1. Study specimens

A total of 103 extant adult avian species comprising 34 orders and 68 families were sampled from the bird collection of the Muséum National d'Histoire Naturelle (MNHN) in Paris (electronic supplementary material, table S1). Taxa were chosen to compile a sample that meets the following criteria: (i) a large range in body mass, (ii) a wide phylogenetic scope representing most major clades, and (iii) a broad spectrum of lifestyles. Based on trait values compiled by Wilman *et al*. [16] and information collected from the literature, each taxon in the present study was assigned to one group of the following ecological categories: diet, foraging and feeding technique (electronic supplementary material, table S2). Diet includes five categories, and taxa are assigned to one dominant diet category [16]. Foraging (10 categories) refers to the substrate where food is taken [16]. Feeding technique (12 categories) refers to the manner in which a food item is obtained [18] with special focus on techniques that involve the neck.

As outlined above, the neck in birds is used for a variety of tasks, but feeding behaviour certainly plays a major role since it is an important factor for the survival of a species. Since categorizing ecology can be difficult and some taxa may not perform exclusively one type of behaviour, the most typical category was selected (refer to electronic supplementary material, text S1: Material and methods—study specimens for more details). The sample includes volant and non-volant birds, with body masses spanning a range of about 34 g to 111 kg. The mean body mass estimates for each of the bird species were obtained from the literature [16].

The hypothesis for the phylogenetic relationships of extant birds considered in the present study is based on molecular data [19]. A consensus topology (strict consensus tree) was created from downloaded samples (100 randomly selected phylogenetic trees from the Global Bird Tree [20]) using the ‘phytools’, ‘ape’, ‘picante’ and ‘geiger’ packages in R [21–25].

### 2.2. Data collection

The total number of CV for each specimen was identified based on observations on vertebral morphology. Potential differences in cervical count in the literature depend on the criteria for assigning the cervicothoracic transition. According to Baumel *et al*. [26], the first thoracic vertebra is defined as the cranial-most vertebra with a complete rib that articulates with the sternum. Therefore, the caudal CV may bear movable ribs, not reaching the sternum [26]. By contrast, Romer [27] identified the cervical count as the number of all vertebrae cranial to the first thoracic vertebra which is defined as having articular facets for freely movable ribs. Here, we follow the latter definition since this makes our dataset more applicable for future analyses including fossil specimens whose preservation may not allow to identify if the rib reached the sternum or not.

The length of the vertebral centrum of all CV was measured using a digital calliper (Mitutoyo; ±0.01 mm) (electronic supplementary material, figure S1a,c,d), except for the atlas (CV1) which does not have a centrum. The total neck length was calculated as the sum of all vertebral lengths and did not include any estimates of the size of the intervertebral discs or intercentrum cartilage. Dividing the cervical vertebral column into two parts indicates the central vertebra. For instance, the total number of vertebrae in the neck is 18 CV in the emu. The central vertebra is CV9. In the case of uneven cervical count, the number was rounded up to the nearest integer. The nandu has 15 CV and the central vertebra is CV8. This allows comparing the length of an individual vertebra across taxa that differ in total cervical count since the central vertebra is usually the longest within the neck (C.B., O.P. and A.A. 2018, personal observation).

The lengths of three long bones of the hindlimb (femur, tibiotarsus and tarsometatarsus) were measured using a digital calliper sensitive to 0.01 mm (electronic supplementary material, figure S1b). The right-side element was measured, unless absent. The length of the bones was measured in a straight line parallel to the long axis of the diaphysis between the borders of the most proximal to the most distal part. The tibiotarsus length did not include prominent crests on the proximal end of the bone. Following Gatesy & Middleton [28], the sum of the lengths of femur, tibiotarsus and tarsometatarsus was calculated to obtain the total leg length for each taxon. The forelimb bones were not included in the present study since the measurements would be highly correlated to flight mode (e.g. [29]) and are, thus, not as informative for assessing body size and habitat use [30].

### 2.3. Statistical analyses

After collecting the total number of CV for each taxon, the median value was calculated for all studied birds. In order to explore the phylogenetic distribution of cervical count across birds, the deviation (more or less CV) from the median number of CV was plotted on the avian phylogeny. Since closely related organisms tend to resemble each other for most aspects of the phenotype [31], the phylogenetic signal was statistically tested. Using the function ‘phylosig’ (‘phytools’ package in R), the Blomberg's *K* was calculated to test whether the same cervical count is present in related taxa more frequently than expected by Brownian motion [32]. A value of *K* > 1 indicates a strong phylogenetic signal, while a value of *K* close to zero indicates a weak phylogenetic signal [32]. Note, however, that other evolutionary models (e.g. Ornstein–Uhlenbeck) may provide a better fit of the data distribution across the phylogeny if adaptive peaks are present in the data [33,34].

In order to assess scaling patterns among individual vertebral length, total neck length, hindlimb elements and body mass, a series of regression analyses was performed. The strength of correlation between the obtained variables was determined using the coefficient of determination (*R*²) values and statistical significance of those correlations (*p*-value) from ordinary least-squares (OLS) regression [35]. To test if the slope of the regression equals isometry (*a* = 1), the function ‘slope.test’ (‘smatr’ package in R) with ‘OLS’ line fitting method was applied [36]. For all analyses, we used the natural log (ln) of these data to mitigate the effects of extreme outliers on regression coefficients [35]. We also tested for potential phylogenetically driven non-independence of the regressed variables using the phylogenetically independent contrasts (PICs) [37,38]. This method estimates and tests the regression between two variables while correcting for the non-independence of data points resulting from phylogeny. It assumes that the traits follow a Brownian motion model of evolution with unchanging rates through time and along all branches of the tree [39]. We used the ‘pic’ function (‘ape’ package in R) to compute the PICs of each variable [24] and performed the same series of regression analyses as with the original data.

To test if cervical count and neck length are independent of ecological category, Spearman's rank correlation tests were performed using the function ‘cor.test’ in R.

## 3. Results

### 3.1. Number of cervical vertebrae

**Figure 1.**
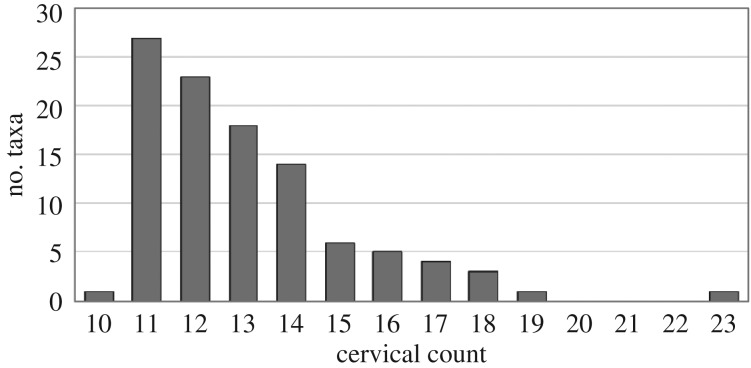
Cervical count. The histogram displays the distribution of the cervical count per taxon (total number of taxa = 103). Note that there is only one taxon having 10 CV and only one taxon having 23 CV. There are no birds in the sample having 20, 21 or 22 CV.

The median value of cervical count for all studied birds is 13 (electronic supplementary material, table S3). The minimum number of 10 vertebrae in the neck is represented only by one taxon in our dataset, blue-and-yellow macaw (*Ara ararauna*). Similarly, there is only one taxon that displays the maximum number of 23 vertebrae in the neck: the mute swan (*Cygnus olor*). There are no birds in the sample that have 20, 21 or 22 CV (figure 1).

**Figure 2.**
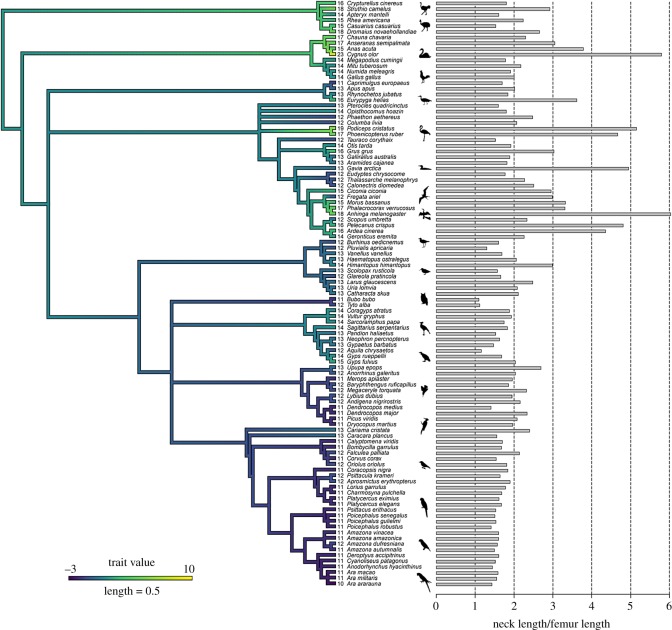
Cervical count and phylogeny. The molecular phylogeny is based on Hackett *et al*. [19]. The branches of the tree are coloured according to increases and decreases in cervical count from the median value of 13 vertebrae in the neck. Numbers indicate the total number of CV per taxon. Grey bars indicate neck length in relation to femur length (which is an indicator for body size).

Our results revealed a strong phylogenetic signal (Blomberg's *K* = 1.500986, *p* = 0.001) indicating that closely related species tend to have similar numbers of CV (figure 2). Palaeognathae and Galloanseriformes display the highest cervical counts, whereas Australaves (including seriamas, falcons, parrots and passerines) have the lowest cervical counts (electronic supplementary material, table S3).

There is no significant relationship between total number of CV and ecological categories across the studied birds (for diet: d.f. = 101, *p*-value = 0.98, *p*-value(PIC) = 0.98; foraging: d.f. = 101, *p*-value = 0.33, *p*-value(PIC) = 0.33; feeding technique: d.f. = 101, *p*-value = 0.07, *p*-value(PIC) = 0.07).

### 3.2. Neck length and body size

Femur length scales isometrically with body mass^1/3^, which is a standard indicator of body size (table 1). The relation between neck length and femur length is isometric as well (table 1), indicating that the length of the cervical vertebral column increases as body size increases. Comparing the scaling coefficients with the phylogenetically corrected data shows that non-independence does not significantly alter the scaling patterns obtained from the raw data (table 1).

**Table 1.** Regression analysis. The relationship between the obtained log-transformed variables (body mass, femur length, tibiotarsus length, tarsometatarsus length, total leg length, neck length and central vertebra length) is quantified with the slope (*a*) of the linear regression: white, isometry (*a* = 1); grey, negative allometry (*a* < 1). The strength of correlation was determined using the coefficient of determination (*R*^2^) values and statistical significance of those correlations (*p*-value) from OLS regression. Significance levels of *p*-values are indicated by asterisks. A regression using the phylogenetic independent contrasts (PICs) calculated with the phylogeny was employed to control for potential non-independence of the data.

variables		linear regression		PIC	
*x*	*y*	*a*	*R*²	*a*	*R*²
body mass^1/3^	femur length	0.97	0.84***	0.86	0.64***
body mass^1/3^	total leg length	1.09	0.79***	0.97	0.58***
femur length	neck length	1.09	0.78***	0.95	0.71***
tibiotarsus length	neck length	1.01	0.85***	0.95	0.82***
tarsometatarsus length	neck length	0.74	0.76***	0.65	0.65***
total leg length	neck length	0.97	0.84***	0.95	0.81***
femur length	central vertebra length	0.91	0.80***	0.93	0.66***
tibiotarsus length	central vertebra length	0.84	0.87***	0.85	0.83***
tarsometatarsus length	central vertebra length	0.61	0.77***	0.64	0.73***

*p < 0.05.

**p < 0.01.

***p < 0.001.

**Figure 3.**
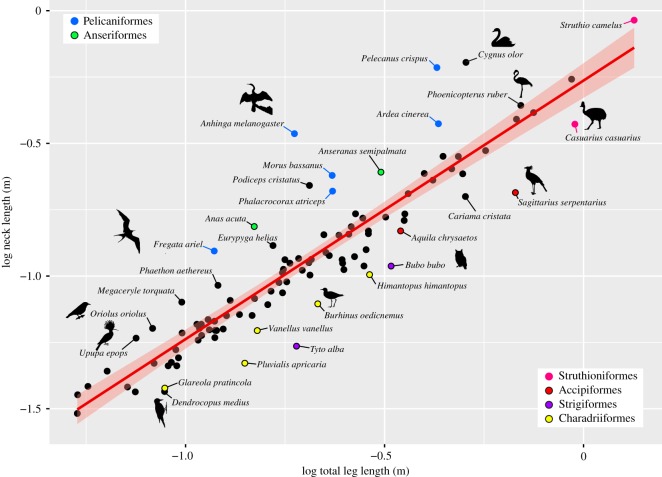
Scaling relationship between neck length and total leg length in birds. In most birds, neck length scales isometrically (*a* = 0.97, *r*² = 0.84, *p* < 0.001). Taxa that have a relatively longer neck lie above the linear regression line (red line with 95% confidence interval), whereas taxa with a relatively shorter neck are below it.

Body size-corrected neck length (regressed against femur length as indicator for body size) varies in the sample (figure 3). The mean neck ratio is 2.18 as represented by the razor-billed curassow (*Mitu tuberosum*). The shortest neck ratio has been found in owls (*Bubo bubo* and *Tyto alba*). The plover (*Pluvialis apricaria*) and the eagle (*Aquila chrysaetos*) also have relatively short necks in relation to femur length. The longest neck ratio has been detected in the darter (*Anhinga melanogaster*) and the swan (*C. olor*) (figure 3). Exceptional long necks in relation to femur length have also been found in the grebe (*Podiceps cristatus*) and the flamingo (*Phoeni copterus ruber*), the loon (*Gavia arctica*), the pelican (*Pelecanus crispus*) and the heron (*Ardea cinerea*).

### 3.3. Neck length in relation to total leg length

In accordance with a previous report [40], the total leg length is also an accurate indicator of body mass in birds. The sum of the lengths of femur, tibiotarsus and tarsometatarsus (total leg length) is proportional to body mass^1/3^, which is a standard indicator of body size (table 1).

In the majority of sampled taxa, neck length showed an isometric scaling in relation to total leg length (*a* = 0.97, *r*² = 0.84, *p* < 0.001) (figure 3). Outliers include taxa that have a relatively longer neck (above linear regression line) and a relatively shorter neck (below linear regression line). Pelagic specialists and birds that forage below or around the water surface (Pelicaniformes, Anseriformes) tend to have a disproportional long neck, whereas many birds that forage on the ground (Charadriiformes) tend to have a relatively short neck (figure 3).

There is no significant relationship between neck length and ecological categories across the studied birds. Yet, there is a trend between neck length and feeding technique (electronic supplementary material, figure S2). Birds that are especially adapted to crack open seeds or nuts (cracker) tend to have an intermediate short neck in relation to their leg length. Ripper include birds with the shortest necks. Browser, plunge-diver and striker are rather variable in neck length, but include the longest-necked taxa.

### 3.4. Vertebra length

**Figure 4.**
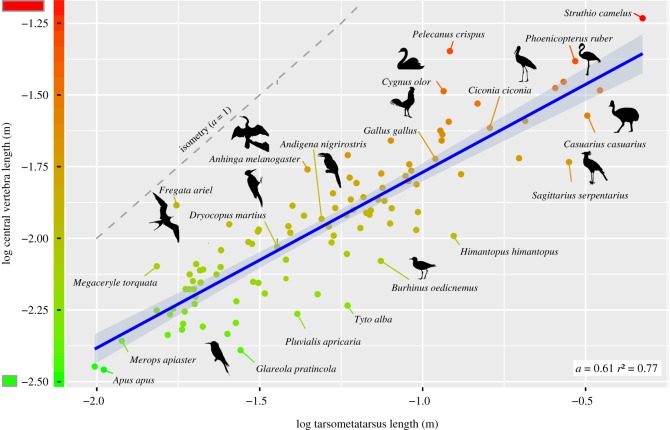
Scaling relationship between vertebra length and tarsometatarsus length in birds. Central vertebra length scales with strong negative allometry (blue line with 95% confidence interval) in relation to tarsometatarsus length. The theoretical isometric line (*a* = 1) is given in grey.

Central vertebra length shows moderate negative allometry in relation to femur length and tibiotarsus length (table 1). It scales with strong negative allometry in relation to tarsometatarsus length (table 1 and figure 4).

## 4. Discussion

### 4.1. Cervical count and phylogeny

The investigation of the cervical vertebral column in a large sample of birds differing in size, taxonomic lineage and lifestyle revealed that variation in cervical count is related to the phylogenetic relatedness of species. This indicates the presence of constraints imposed by evolutionary heritage [41]. However, focusing on individual taxonomic groups within the bird sample reveals taxon-specific variation of the number of CV. For instance, within Galloanseriformes, the range of variation in cervical count is from 14 to 23 with pelagic specialists having a higher number of vertebrae in the neck (figure 2). Correspondingly, within Aequornithes, the cervical count ranges from 12 to 18 with pelagic specialists having a higher number of vertebrae in the neck. Within Accipitriformes, the range of variation in cervical count is from 12 to 15 and reflects ecological specializations. Vultures, as obligate scavengers, have occupied a special ecological niche by exclusively feeding on carrion. However, competition among sympatric vultures led to ecological differences such as preference of certain types of food from a carcass [42,43]. The ‘short-necked’ (13 CV) cinereous vulture (*Aegypius monachus*) is a typical ‘ripper’ who feeds primarily on tough skin and hide of a carcass. During feeding, it tears off tendons from bones and pieces of skin [44]. By contrast, the ‘long-necked’ (15 CV) griffon vulture (*Gyps fulvus*) is a typical ‘gulper’ who feeds primarily on the softer viscera [44]. These differences in feeding ecology have been correlated to differences in skull shape and the present study suggests that they correlate to the number of CV as well. Similar relaxed constraints have been reported in taxa with ‘extreme’ ecologies such as marine mammals or reptiles [45]. In summary, the ‘group-specific selection’ (as opposed to ‘clade-wide selection’) may suggest the absence or release of constraints during evolution and appears to be related to ecological differences ([41,45]; this study).

**Figure 5.**
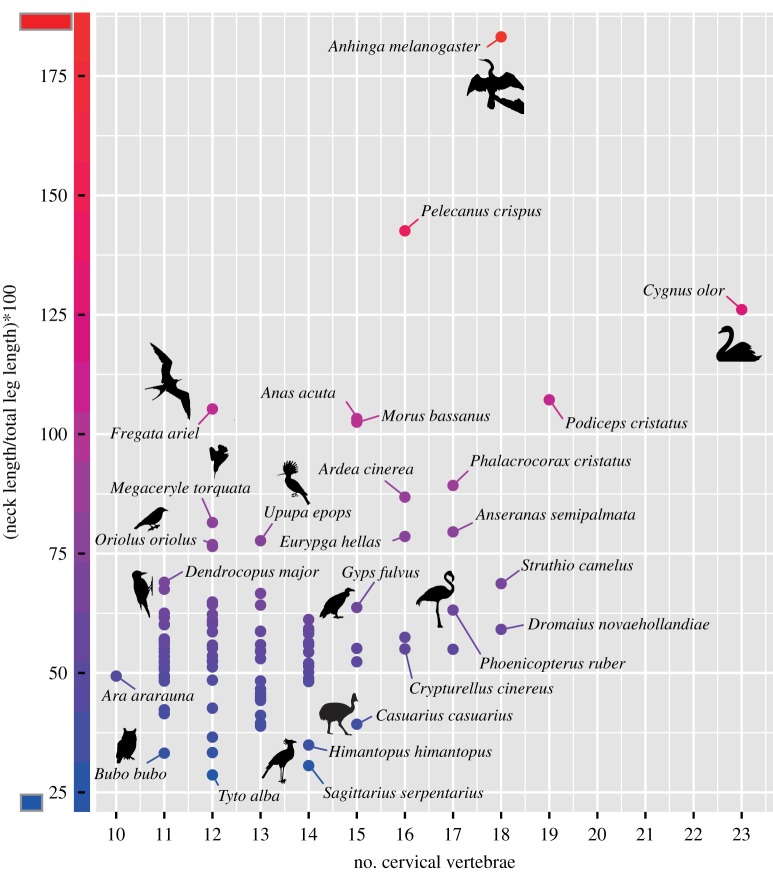
Neck length, vertebra length and cervical count in birds. The same number of CV may form a rather long or short neck in relation to total leg length.

Although it is plausible that a larger number of intervertebral joints generally enhances the mobility in the vertebral column, it is not surprising that overall the cervical count is not significantly correlated with function since vertebral morphology also plays a crucial role in this context. The present study shows that the identical number of CV may form either a relatively short or long neck, indicating that the length of the vertebrae differs between species (figure 5). The dimensions of the vertebrae, notably the length of the vertebral centrum, have considerable impact on the degree of mobility and, thus, function (e.g. [46,47]). For instance, 15 CV form the relatively short neck in the cassowary (*Casuarius casuarius*), whereas the same cervical count is found in the relatively long neck of the heron (*A. cinerea*) (figure 5). The feeding technique in these two taxa is quite different (gleaner versus striker). Thus, cervical count alone is not sufficient to evaluate the function of the neck in birds.

### 4.2. Cervical count and development

The recorded values of cervical count recorded in the present study (electronic supplementary material, table S3) lie within the range of values previously reported in the literature (e.g. [4,48]). Although birds are evidently very variable in cervical count, the majority of the sampled bird taxa (79%) have 11 to 14 CV (figure 1). A mean value of 14.82 CV in birds has also been reported by Müller *et al*. [45]. Surprisingly, a number of 20, 21 or 22 vertebrae in the neck are not represented (figure 1) and the maximum cervical count is only represented by one taxon, *C. olor* (23 CV). This raises the question whether this is due to a sampling bias or if there are any constraints against this cervical count. The literature review revealed that within Palaeognathae, there is fossil evidence for cervical counts of 20–23 vertebrae in the extinct moas (e.g. [49,50]). Furthermore, a range of 17–21 CV has been reported in grebes [51]. Beyond birds, the only other amniote groups that evolved a comparable high number of CV includes the extinct sauropod dinosaurs with a maximum of 18 CV [52] and the extinct plesiosaurs with a maximum of 76 CV [53]. Thus, the lack of a number of 20–22 vertebrae in the neck of birds in the present study is indeed due to a sampling bias. Nevertheless, it is interesting to note that a cervical count of more than 20 is rather rare in terrestrial amniotes. A potential constraint in cervical count may be linked to the process of somitogenesis which is responsible for the species-specific number of somites (i.e. segments that later develop into vertebrae) [54]. In principle, the process is an open-ended system and, thus, total somite number can vary widely between vertebrate species [55]. The comparative analysis of early embryonic development between the emu (*Dromaius novaehollandiae*, 18 CV) and the chicken (*Gallus gallus*, 14 CV) revealed that more somites are generated in the palaeognath embryo than in the neognath embryo at the same developmental stage [56]. In this regard, it would be highly interesting to study the embryonic development of the swan (e.g. *C. olor*, 23 CV) in order to assess if heterochrony in somitogenesis rate is responsible for the increase in segment number. In snakes which have more than 300 segments, it has been shown that an increase in the number of somites is the result of an accelerated rate of somitogenesis relative to body axis growth on the one hand, and of sustained paraxial mesoderm growth (higher number of cell generations) on the other hand [57]. Furthermore, measurements of the somite size revealed that snake somites are at least three times smaller than mouse or chicken somites [57]. The correlation between the length of the somite and the number of cells in the somite [58] suggests that an indefinite increase in the speed of somitogenesis associated with a decrease in somite size is not possible. Embryonic manipulation experiments involving the removal of somites revealed that a deficiency of somitic material can produce vertebral anomalies in chickens [59]. Thus, there may be a trade-off between increased number of somites (and hence vertebrae) and size of somites (amount of cells forming the vertebrae) which could lead to a developmental constraint against extremely high cervical counts. Strikingly, the present results showed a strong trend between cervical count and individual vertebral length: the higher the number of vertebrae in the neck, the shorter the central cervical vertebra in relation to total neck length (electronic supplementary material, figure S3).

### 4.3. Body size does not constrain neck length

Overall, the detected relationship between neck length and body size in birds (isometry, table 1) is in contrast with mammals in which neck length tends to decrease with increasing body size [60]. The negative allometry in mammals results from the observation that the weight of the head increases faster (power of three) than the stress-resisting cross-sectional area of the neck (power of two) [61–64]. Birds, however, are characterized by a high skeletal pneumatization and they have relatively small heads [65,66]. Even taxa with very large beaks, such as the toucan, are constructed as lightweight [67]. Consequently, the head does not limit neck length in birds.

### 4.4. Neck length and ecology

Although the present study did not detect a significant correlation between size-corrected neck length and ecology, the results suggest a gradual trend towards shorter necks in terrestrial taxa and longer necks in aquatic taxa. In part, this may be a side effect of generally short legs in swimming taxa, but nevertheless an exceptional short or long neck tends to be associated with a specialized foraging and feeding technique. The rather long necks in several (semi-)aquatic birds such as ducks (*Anas acuta*) and swans (*C. olor*) (figure 2) allow them to efficiently feed below the level of their feet (i.e. underwater). Plunge-diving specialists such as the frigatebird (*Fregata ariel*), the gannet (*Morus bassanus*) and even the kingfisher (*Megacerycle torquata*) tend to have a rather long neck as well. This may be correlated to the mechanical demands for supporting the high forces that the bird experience during the impact with the water [68].

Interestingly, terrestrial probing birds such as the kiwi (*Apteryx mantelli*) or the Eurasian woodcock (*Scolopax rusticola*) have relatively short necks. However, this is combined with a long beak which allows them to forage on the ground in woodlands [69]. By contrast, semi-aquatic probing birds, such as the Eurasian oystercatcher (*Haematopus ostralegus*) or the black-winged stilt (*Himantopus himantopus*), tend to have a long beak in combination with rather a long neck. This appears to be advantageous for foraging on coastal intertidal flats [70].

Woodpeckers typically peck holes into the wooden substrate in order to feed on invertebrates. Nevertheless, different species occupy different feeding habitat niches and, thus, differ in pecking behaviour with more arboreal (e.g. *Dendrocopus major*) or less arboreal (e.g. *Picus viridis*) taxa [71,72]. All sampled woodpeckers have a relatively short neck. This may be linked to the morphofunctional constraints imposed by the pecking behaviour, but a phylogenetic signal cannot be excluded because there are no other birds that convergently evolved a woodpecker-like ecology. From a biomechanical point of view, it is not surprising that taxa whose neck experiences very high forces are generally rather short. The psittaciform birds have the shortest necks in our sample and many of these animals use their neck during arboreal locomotion [13].

### 4.5. Correlated evolution between neck length and leg length

Differences in the overall body plan of birds are mainly due to differences in the hindlimb module (i.e. length of the hindlimb segments) [28,30,73–75]. The extreme functional divergence of the fore- and hindlimbs has resulted in reduced covariation between limbs (decoupling of the forelimb and hindlimb module), allowing increased independent variation in serially homologous structures [3,76]. In contrast with this weak integration, a related evolution between pectoral and pelvic muscle mass [77], between pectoral limb size, centre of mass position and hindlimb posture [78] as well as between sternal keel and ilium length [79] has been found in birds. Although the presence of sampling bias (i.e. overrepresentation of certain taxa) can potentially impact the findings, the present study detected a correlated evolution of neck length and total leg length in extant birds, suggesting a strong integration between the cervical and pelvic module. Indeed, in giraffes, it is not the long neck alone, but the overall vertical elongation of the giraffe body form (including the elongate legs) that allows them to gain a foraging advantage by browsing above the reach of smaller browsers [80]. In birds, longer legs may necessitate a longer neck allowing the head to reach the ground. Vital tasks that birds perform on or near the ground level include food intake, drinking and feeding their offspring [5,81]. In contrast with other amniotes, birds keep their centre of mass over the foot using a crouched hindlimb posture [78,82]. In principle, a bipedal, relatively short-necked animal is able to reach the ground level by rotating the trunk in relation to the hindlimbs as may have been the case in non-avian theropods. However, the latter had a counterbalancing tail which is reduced in birds (e.g. refer to Grossi *et al*. [83] for the effects of a tail altering the centre of mass in chickens). Therefore, it appears that avian neck length is important in relation to hindlimb length and there may be a coelongation between both modules.

### 4.6. Vertebra length and tarsometatarsus length

The present study found a strong negative allometry between central vertebral length and tarsometatarsus. However, the correlation is not very strong (raw *R*^2^ = 0.77 and phylogenetically corrected *R*^2^ = 0.73; table 1) which may be due to the relatively high variability in tarsometatarsus length as opposed to femur and tibiotarsus length (electronic supplementary material, figure S4). It has been previously shown that, among the three hindlimb bones, the tarsometatarsus presents the greatest morphological disparity across species (e.g. [84,85]). The relation between vertebral length and tarsometatarsus length may potentially support a reported trade-off between quantitative design (i.e. relative volumes of bone) and maximum rates of avian post-hatching growth [86]. In general, the limbs in vertebrates and in particular the metapodial bones grow faster than the rest of the body (i.e. allometric growth) [87–89]. The tarsometatarsus bone is one of the longest and fastest growing in the body of birds [90]. It has been suggested that the size of organs with vital functions may influence the size of other organs [87]. Thus, the pattern of development of the tarsometatarsus may potentially limit the relative growth of the vertebrae resulting in relatively shorter vertebrae. Yet, caution must be exerted when interpreting the observed negative allometry between vertebrae and leg bone. It would be highly interesting to substantiate this observation by further ontogenetic and developmental studies that investigate the actual growth rate of these skeletal elements.

## 5. Conclusion

Within amniotes, birds show comparatively little diversity in overall ‘body plan’. High interspecific variability is evident in the length proportions of the hindlimb bones (e.g. [30]) as well as in the number of CV ([45]; this study). However, cervical count in birds is strongly related to phylogeny, with only some specialists having an exceptional number of vertebrae in the neck. Furthermore, neck length scales isometrically with total leg length indicating a relative dependence between both modules. The strong integration between the cervical and pelvic module in birds is in contrast with the weak integration between pectoral and pelvic module. This may be the result of the evolution of a functionally constrained forelimb due to the evolution of powered flight which is compensated by a functionally versatile neck.

In future analyses, we aim to study the covariation between cranial and cervical module since we expect them to be highly integrated, yet we lack knowledge in this regard. This involves also to put into relation the neck length to the length of the beak. The present results suggest two trends: a short neck combined with a long beak in terrestrial probing bird and a rather long neck in combination with a long beak in semi-aquatic probing birds. Further analyses will illuminate the covariation between beak and neck.

## Supplementary Material

Supplementary Material

## Data accessibility

Data relating to this work are provided in the electronic supplementary material.
