# Supplementary material for: Correlated evolution of neck length and leg length in birds
Source: R Soc Open Sci. 2019 May 8;6(5):181588. doi: 10.1098/rsos.181588 (PMC6549945; doi:10.1098/rsos.181588)
Supplement: Supplementary Material [file rsos181588supp1.docx]

**SUPPLEMENTARY MATERIAL**

**Böhmer et al.: “Correlated Evolution of Neck Length and Leg Length in Birds”**

**Content:**

**Supplementary text 1**: Material and methods - Study specimens

**Figure S1**: Measurements.

**Figure S2**: Neck length and feeding technique in birds.

**Figure S3**: Neck length, vertebra length, and cervical count in birds.

**Figure S4**: Variation in leg bone length.

**Table S1**: Data.

**Table S2**: Explanation of the ecological categories.

**Table S3**: Cervical count in birds.

**Supplementary material references**

**Supplementary text 1**: Material and methods - Study specimens

The neck in birds is used for a variety of tasks, but feeding behavior certainly plays a major role since it is an important factor for the survival of a species. Since categorizing ecology can be difficult and some taxa may not perform exclusively one type of behavior, the most typical category was selected. For instance, the southern rockhopper penguin (*Eudyptes chrysocome*) feeds prevalently on invertebrates (diet) below the water surface (foraging) and catches its food items by pursuit diving (feeding technique). In contrast, the griffon vulture (*Gyps fulvus*) scavenges prevalently on vertebrates and fish (diet) on the ground (foraging) and gets its food items by ripping (feeding technique).

**Figure S1**: **Measurements**. Photographs illustrate the taken measurements on (**a**) the cervical vertebrae (ce = centrum length; *Gavia arctica*, MNHN MO-AC 1992-9, in dorsal view) and (**b**) three long bones of the hindlimb (fe = femur, ti = tibiotarsus, ta = tarsometatarsus; *Otis tarda*, MNHN 1875-784, in anterior view). The functional vertebral length was measured using (**c, d**) a digital caliper with larger or smaller accessory pins depending on the size of the vertebrae (adapted from [Taylor and Wedel [1](#_ENREF_1)]). Abbreviation: MNHN = Muséum National d’Histoire Naturelle in Paris.


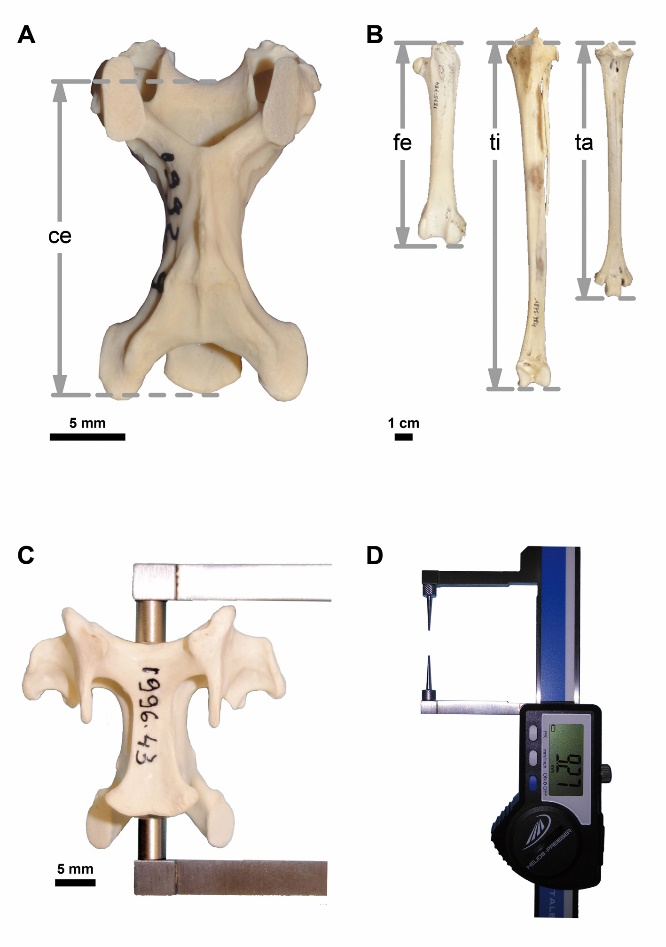


**Figure S2**: **Neck length and feeding technique in birds**. There appears to be a trend towards having a rather short neck in relation to total leg length in cracker, forager, gleaner, and ripper. The other feeding technique categories are more variable, but browser, plunge-diver, and striker include the longest-necked taxa. Refer to table S2 for explanation of ecological categories.


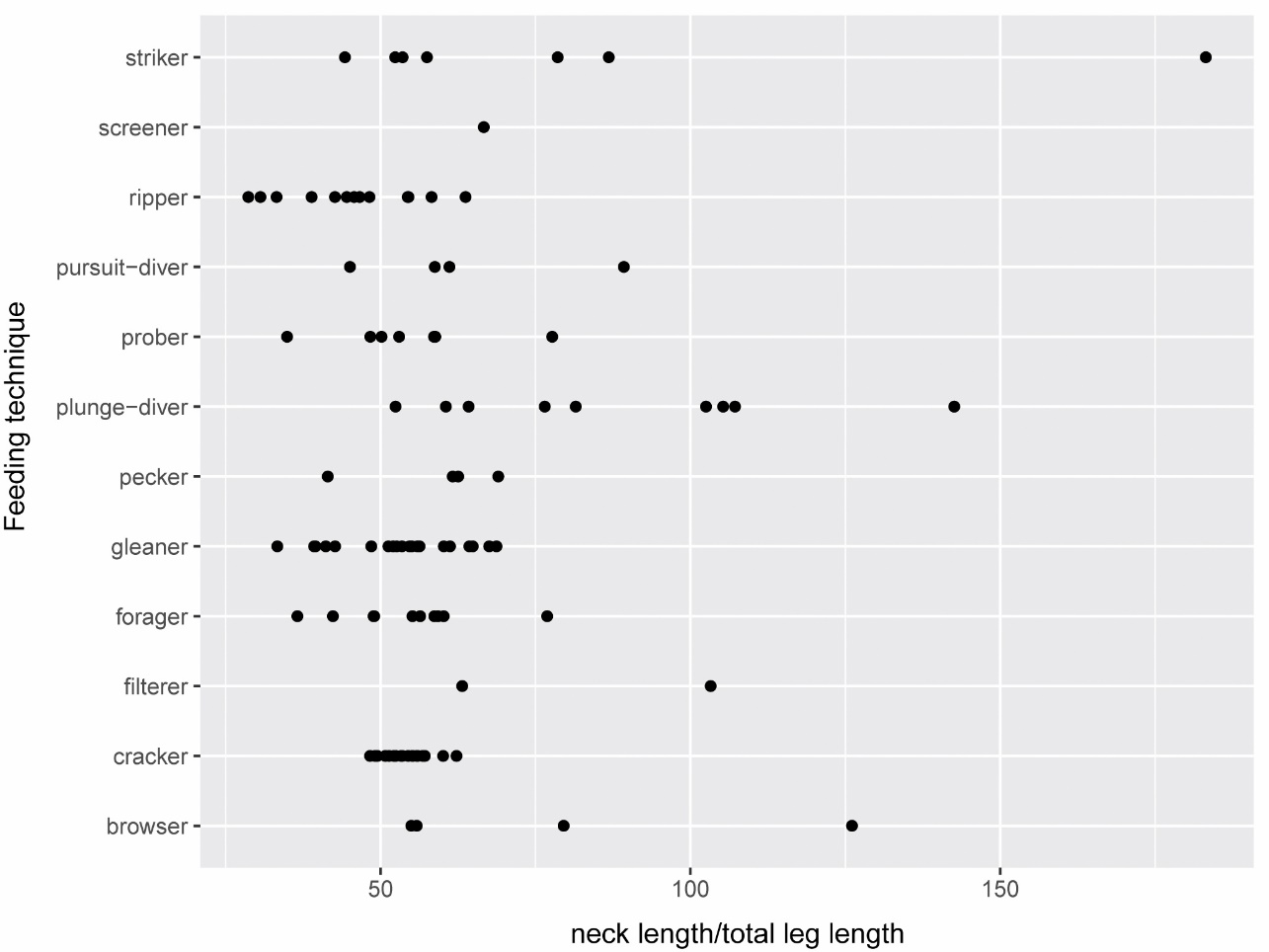


**Figure S3**: **Neck length, vertebra length, and cervical count in birds**. The higher the cervical count, the shorter the central vertebra in relation to neck length.


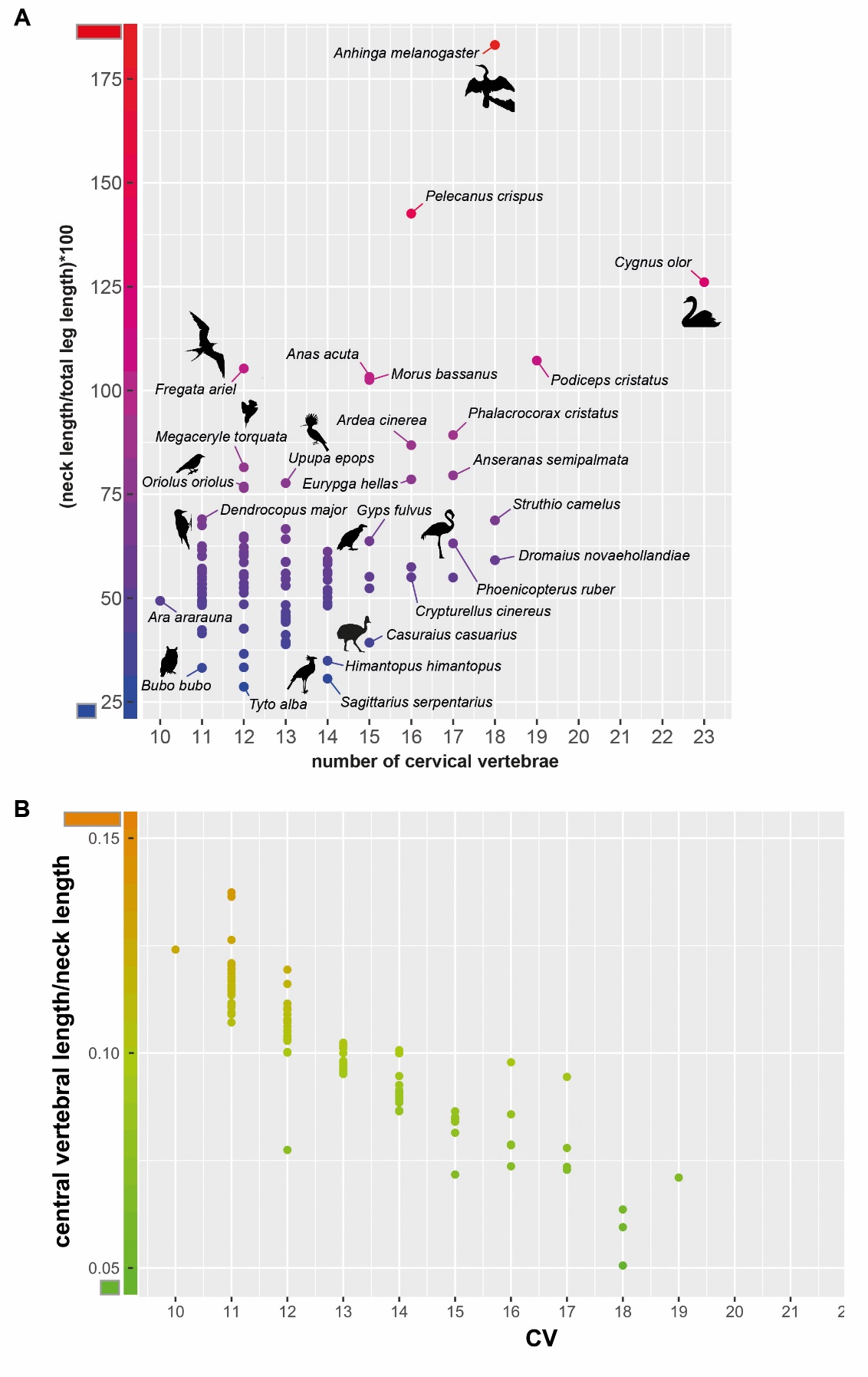


**Figure S4**: **Variation in leg bone length.** Boxplot showing the variance of the obtained log-transformed variables (femur lengt, tibiotarsus length, tarsometatarsus length).


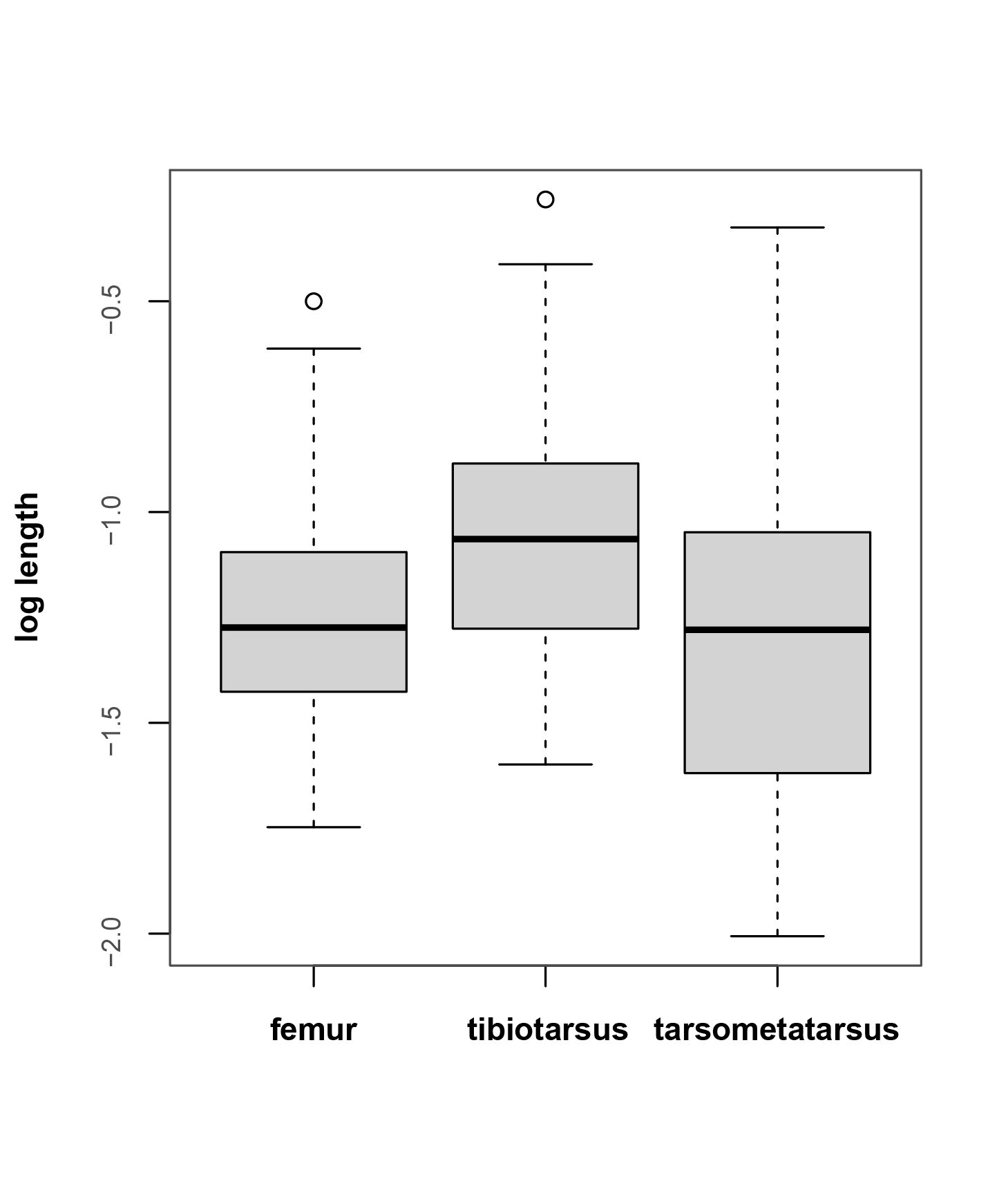


**Supplementary Table S1: Data.** A total of 103 extant avian species comprising 34 orders and 68 families were sampled from the bird collection of the Muséum National d’Histoire Naturelle in Paris. Taxa were chosen to comprise a large range in body mass, a wide phylogenetic scope representing most major clades, and a broad spectrum of lifestyles. Based on trait values compiled by Wilman, H., Belmaker, J., et al. (2014) and information collected from the literature, each taxon in the present study was assigned to one group of the following ecological categories: diet, foraging, body mass, and feeding technique (Refer to table 1 for explanation of the categories). Diet includes five categories and taxa are assigned to one dominant diet category (Wilman et al. 2014). Foraging (10 categories) refers to the substrate where food is taken (Wilman et al. 2014). Body mass is classified into six body mass bins. Feeding technique (12 categories) refers to the manner in which a food item is obtained (de Graaf et al. 1985) with special focus on techniques that involve the neck.

| **Order** | **Taxon** | **Diet** | **Forarging** | **Body mass bin** | **Feeding technique** |
| --- | --- | --- | --- | --- | --- |
| Accipitriformes | *Aquila chrysaetos* | VertFishScav | ForStrat.ground | 4000-12000 | ripper |
| Accipitriformes | *Coragyps atratus* | VertFishScav | ForStrat.ground | 1000-4000 | cracker |
| Accipitriformes | *Gypaetus barbatus* | VertFishScav | ForStrat.ground | 4000-12000 | ripper |
| Accipitriformes | *Gyps fulvus* | VertFishScav | ForStrat.ground | 4000-12000 | ripper |
| Accipitriformes | *Gyps rueppellii* | VertFishScav | ForStrat.ground | 4000-12000 | ripper |
| Accipitriformes | *Neophron percnopterus* | VertFishScav | ForStrat.ground | 1000-4000 | ripper |
| Accipitriformes | *Pandion haliaetus* | VertFishScav | ForStrat.wataroundsurf | 1000-4000 | ripper |
| Accipitriformes | *Sagittarius serpentarius* | Invertebrate | ForStrat.ground | 4000-12000 | ripper |
| Accipitriformes | *Sarcoramphus papa* | VertFishScav | ForStrat.ground | 1000-4000 | ripper |
| Accipitriformes | *Vultur gryphus* | VertFishScav | ForStrat.ground | 4000-12000 | ripper |
| Anseriformes | *Anas acuta* | PlantSeed | ForStrat.watbelowsurf | 100-1000 | filterer |
| Anseriformes | *Anseranas semipalmata* | PlantSeed | ForStrat.ground | 1000-4000 | browser |
| Anseriformes | *Chauna chavaria* | PlantSeed | ForStrat.ground | 1000-4000 | browser |
| Anseriformes | *Cygnus olor* | PlantSeed | ForStrat.watbelowsurf | 4000-12000 | browser |
| Apodiformes | *Apus apus* | Invertebrate | ForStrat.aerial | 10-40 | screener |
| Apterygiformes | *Apteryx mantelli* | Invertebrate | ForStrat.ground | 1000-4000 | prober |
| Bucerotiformes | *Anorrhinus galeritus* | FruiNect | ForStrat.midhigh | 1000-4000 | gleaner |
| Bucerotiformes | *Upupa epops* | Invertebrate | ForStrat.ground | 40-100 | prober |
| Caprimulgiformes | *Caprimulgus europaeus* | Invertebrate | ForStrat.understory | 40-100 | gleaner |
| Cariamiformes | *Cariama cristata* | Invertebrate | ForStrat.ground | 1000-4000 | gleaner |
| Casuariiformes | *Casuarius casuarius* | FruiNect | ForStrat.ground | >12000 | gleaner |
| Casuariiformes | *Dromaius novaehollandiae* | Omnivore | ForStrat.understory | >12000 | forager |
| Charadriiformes | *Burhinus oedicnemus* | Omnivore | ForStrat.ground | 100-1000 | forager |
| Charadriiformes | *Catharacta skua* | VertFishScav | ForStrat.wataroundsurf | 1000-4000 | ripper |
| Charadriiformes | *Glareola pratincola* | Invertebrate | ForStrat.ground.under.mid | 40-100 | gleaner |
| Charadriiformes | *Haematopus ostralegus* | Invertebrate | ForStrat.ground | 100-1000 | prober |
| Charadriiformes | *Himantopus himantopus* | Invertebrate | ForStrat.wataroundsurf | 100-1000 | prober |
| Charadriiformes | *Larus glaucescens* | VertFishScav | ForStrat.wataroundsurf | 1000-4000 | plunge-diver |
| Charadriiformes | *Pluvialis apricaria* | Invertebrate | ForStrat.ground | 100-1000 | gleaner |
| Charadriiformes | *Scolopax rusticola* | Invertebrate | ForStrat.ground | 100-1000 | prober |
| Charadriiformes | *Uria lomvia* | VertFishScav | ForStrat.watbelowsurf | 100-1000 | pursuit-diver |
| Charadriiformes | *Vanellus vanellus* | Invertebrate | ForStrat.ground | 100-1000 | gleaner |
| Ciconiiformes | *Ciconia ciconia* | VertFishScav | ForStrat.ground | 1000-4000 | striker |
| Columbiformes | *Columba livia* | PlantSeed | ForStrat.ground | 100-1000 | gleaner |
| Coraciiformes | *Baryphthengus ruficapillus* | Invertebrate | ForStrat.ground | 100-1000 | gleaner |
| Coraciiformes | *Megaceryle torquata* | VertFishScav | ForStrat.watbelowsurf | 100-1000 | plunge-diver |
| Coraciiformes | *Merops apiaster* | Invertebrate | ForStrat.midhigh | 40-100 | gleaner |
| Eurypygiformes | *Eurypyga helias* | VertFishScav | ForStrat.ground | 100-1000 | striker |
| Eurypygiformes | *Rhynochetos jubatus* | VertFishScav | ForStrat.ground | 100-1000 | striker |
| Falconiformes | *Caracara plancus* | VertFishScav | ForStrat.ground | 1000-4000 | ripper |
| Galliformes | *Gallus gallus* | Omnivore | ForStrat.ground | 100-1000 | forager |
| Galliformes | *Megapodius cumingii* | Invertebrate | ForStrat.ground | 1000-4000 | gleaner |
| Galliformes | *Mitu tuberosum* | FruiNect | ForStrat.ground | 1000-4000 | gleaner |
| Galliformes | *Numida meleagris* | PlantSeed | ForStrat.ground | 1000-4000 | forager |
| Gaviiformes | *Gavia arctica* | VertFishScav | ForStrat.wataroundsurf | 1000-4000 | pursuit-diver |
| Gruiformes | *Aramides cajanea* | Omnivore | ForStrat.ground | 100-1000 | gleaner |
| Gruiformes | *Gallirallus australis* | Omnivore | ForStrat.ground | 100-1000 | forager |
| Gruiformes | *Grus grus* | PlantSeed | ForStrat.ground | 4000-12000 | striker |
| Musophagiformes | *Tauraco corythaix* | FruiNect | ForStrat.midhigh | 100-1000 | gleaner |
| Opisthocomiformes | *Opisthocomus hoazin* | PlantSeed | ForStrat.midhigh | 100-1000 | browser |
| Otidiformes | *Otis tarda* | Omnivore | ForStrat.ground | 4000-12000 | forager |
| Passeriformes | *Bombycilla garrulus* | Omnivore | ForStrat.under.mid | 40-100 | forager |
| Passeriformes | *Calyptomena viridis* | FruiNect | ForStrat.understory | 40-100 | gleaner |
| Passeriformes | *Corvus corax* | VertFishScav | ForStrat.ground | 100-1000 | forager |
| Passeriformes | *Falculea palliata* | Invertebrate | ForStrat.midhigh | 100-1000 | prober |
| Passeriformes | *Oriolus oriolus* | Omnivore | ForStrat.canopy | 40-100 | forager |
| Pelecaniformes | *Ardea cinerea* | VertFishScav | ForStrat.wataroundsurf | 1000-4000 | striker |
| Pelecaniformes | *Geronticus eremita* | Omnivore | ForStrat.ground | 1000-4000 | prober |
| Pelecaniformes | *Pelecanus crispus* | VertFishScav | ForStrat.watbelowsurf | 4000-12000 | plunge-diver |
| Pelecaniformes | *Scopus umbretta* | VertFishScav | ForStrat.wataroundsurf | 100-1000 | striker |
| Phaethontiformes | *Phaethon aethereus* | VertFishScav | ForStrat.watbelowsurf | 100-1000 | plunge-diver |
| Phoenicopteriformes | *Phoenicopterus ruber* | Omnivore | ForStrat.wataroundsurf | 1000-4000 | filterer |
| Piciformes | *Andigena nigrirostris* | Omnivore | ForStrat.canopy | 100-1000 | forager |
| Piciformes | *Dendrocopos major* | Omnivore | ForStrat.under.mid.canopy | 40-100 | pecker |
| Piciformes | *Dendrocopos medius* | Invertebrate | ForStrat.under.mid.canopy | 40-100 | pecker |
| Piciformes | *Dryocopus martius* | Invertebrate | ForStrat.ground.under.mid | 100-1000 | pecker |
| Piciformes | *Lybius dubius* | FruiNect | ForStrat.midhigh | 40-100 | gleaner |
| Piciformes | *Picus viridis* | Invertebrate | ForStrat.ground | 100-1000 | pecker |
| Podicipediformes | *Podiceps cristatus* | VertFishScav | ForStrat.watbelowsurf | 100-1000 | plunge-diver |
| Procellariiformes | *Calonectris diomedea* | VertFishScav | ForStrat.wataroundsurf | 100-1000 | plunge-diver |
| Procellariiformes | *Thalassarche melanophrys* | Omnivore | ForStrat.wataroundsurf | 1000-4000 | plunge-diver |
| Psittaciformes | *Amazona amazonica* | FruiNect | ForStrat.under.mid.canopy | 100-1000 | cracker |
| Psittaciformes | *Amazona autumnalis* | FruiNect | ForStrat.under.mid.canopy | 100-1000 | cracker |
| Psittaciformes | *Amazona dufresniana* | Omnivore | ForStrat.midhigh | 100-1000 | cracker |
| Psittaciformes | *Amazona vinacea* | PlantSeed | ForStrat.under.mid.canopy | 100-1000 | cracker |
| Psittaciformes | *Anodorhynchus hyacinthinus* | FruiNect | ForStrat.midhigh | 1000-4000 | cracker |
| Psittaciformes | *Aprosmictus erythropterus* | Omnivore | ForStrat.under.mid | 100-1000 | cracker |
| Psittaciformes | *Ara ararauna* | PlantSeed | ForStrat.midhigh | 1000-4000 | cracker |
| Psittaciformes | *Ara macao* | PlantSeed | ForStrat.midhigh | 1000-4000 | cracker |
| Psittaciformes | *Ara militaris* | FruiNect | ForStrat.midhigh | 1000-4000 | cracker |
| Psittaciformes | *Charmosyna pulchella* | FruiNect | ForStrat.canopy | 10-40 | cracker |
| Psittaciformes | *Coracopsis nigra* | FruiNect | ForStrat.under.mid | 100-1000 | gleaner |
| Psittaciformes | *Cyanoliseus patagonus* | PlantSeed | ForStrat.ground | 100-1000 | cracker |
| Psittaciformes | *Deroptyus accipitrinus* | PlantSeed | ForStrat.under.mid.canopy | 100-1000 | cracker |
| Psittaciformes | *Lorius garrulus* | FruiNect | ForStrat.canopy | 100-1000 | cracker |
| Psittaciformes | *Platycercus elegans* | PlantSeed | ForStrat.midhigh | 100-1000 | cracker |
| Psittaciformes | *Platycercus eximius* | PlantSeed | ForStrat.ground.under.mid | 100-1000 | cracker |
| Psittaciformes | *Poicephalus gulielmi* | PlantSeed | ForStrat.midhigh | 100-1000 | cracker |
| Psittaciformes | *Poicephalus robustus* | FruiNect | ForStrat.midhigh | 100-1000 | cracker |
| Psittaciformes | *Poicephalus senegalus* | PlantSeed | ForStrat.midhigh | 100-1000 | cracker |
| Psittaciformes | *Psittacula krameri* | FruiNect | ForStrat.ground.under.mid | 100-1000 | cracker |
| Psittaciformes | *Psittacus erithacus* | Omnivore | ForStrat.canopy | 100-1000 | cracker |
| Pteroclidiformes | *Pterocles quadricinctus* | PlantSeed | ForStrat.ground | 100-1000 | gleaner |
| Rheiformes | *Rhea americana* | Omnivore | ForStrat.ground | >12000 | forager |
| Sphenisciformes | *Eudyptes chrysocome* | Invertebrate | ForStrat.watbelowsurf | 1000-4000 | pursuit-diver |
| Strigiformes | *Bubo bubo* | VertFishScav | ForStrat.ground | 1000-4000 | ripper |
| Strigiformes | *Tyto alba* | VertFishScav | ForStrat.ground | 100-1000 | ripper |
| Struthioniformes | *Struthio camelus* | PlantSeed | ForStrat.ground | >12000 | gleaner |
| Suliformes | *Anhinga melanogaster* | VertFishScav | ForStrat.watbelowsurf | 1000-4000 | striker |
| Suliformes | *Fregata ariel* | VertFishScav | ForStrat.wataroundsurf | 100-1000 | plunge-diver |
| Suliformes | *Morus bassanus* | VertFishScav | ForStrat.watbelowsurf | 1000-4000 | plunge-diver |
| Suliformes | *Phalacrocorax verrucosus* | Omnivore | ForStrat.watbelowsurf | 1000-4000 | pursuit-diver |
| Tinamiformes | *Crypturellus cinereus* | FruiNect | ForStrat.ground | 100-1000 | gleaner |

**Supplementary Table S2: Explanation of the ecological categories.** Each taxon in the present study was assigned to one group of the following categories based on trait values compiled by Wilman et al. (2014) [[2](#_ENREF_2)] and information collected from the literature. Diet includes five categories and taxa are assigned to one dominant diet category [[2](#_ENREF_2)]. Foraging (10 categories) refers to the substrate where food is taken [[2](#_ENREF_2)]. Feeding technique (12 categories) refers to the manner in which a food item is obtained [[3](#_ENREF_3)] with special focus on techniques that involve the neck.

| **Category** | **Trait & description** | | | | |
| --- | --- | --- | --- | --- | --- |
| **Diet** | *FruitNectar* | *PlantSeed* | *VertFishScav* | *Invert* | *Omnivore* |
|  | fruits and nectar | plant and seeds | vertebrates, fish, carrion | invertebrates | omnivore |
| **Foraging** | *watbelow* | *wataround* | *ground* | *understory* | *midhigh* |
|  | below water surface | on or just (<5 inches) below water surface | ground | below 2m in understory in forest, forest edges, bushes or shrubs | mid to high levels in trees or high bushes (2m upward), but below canopy |
| **Foraging** | *ground.under.mid* | *under.mid* | *under.mid.canopy* | *canopy* | *aerial* |
|  | ground, understory, and midhigh | understory and midhigh | understory, midhigh, and canopy | in or just above (from) tree canopy | well above vegetation or any structures |
| **Feeding technique** | *browser* | *cracker* | *filterer* | *forager* | *gleaner* |
|  | feeds on plants | cracks open seeds or nuts | filters food items from water | takes almost any food items encountered upon the substrate | selects particular food items from the substrate |
| **Feeding technique** | *pecker* | *plunge-diver* | *prober* | *pursuit-diver* | *ripper* |
|  | drilles holes into trees | plunges from air into water | inserts the bill into the substrate | dives from water surface for underwater food | tears prey into pieces |
| **Feeding technique** | *screener* | *striker* |  |  |  |
|  | flies with bill open and screens prey from air | straightens neck to strike with bill very fast |  |  |  |

**Supplementary Table S3: Cervical count in birds**. The present study includes a sample of 103 extant avian species comprising 34 orders and 68 families. They are pooled into seven taxonomic groups. The group “other Neoaves” includes all sampled neoavian birds excluding Aequornithes, Charadriiformes, Telluraves and Australaves.

| **Taxonomic group** | **number of taxa** | **total number of cervical vertebrae** | | |
| --- | --- | --- | --- | --- |
|  |  | **mean** | **max.** | **min.** |
| Aves | 103 | 13 | 23 | 10 |
| Palaeognathae | 6 | 16 | 18 | 14 |
| Galloanseriformes | 8 | 16 | 23 | 14 |
| other Neoaves | 15 | 14 | 19 | 11 |
| Aequornithes | 13 | 14 | 18 | 12 |
| Charadriiformes | 10 | 13 | 14 | 12 |
| Telluraves | 24 | 13 | 15 | 11 |
| Australaves | 27 | 11 | 13 | 10 |

**Supplementary Table S4: Obtained data**. ID = specimen identficiation number. MNHN ZO = Muséum National d’Histoire Naturelle Paris Zoological Collection; BMNH = British Museum of Natural History London. Mean body mass (BM) estimates for each of the bird species were obtained from the literature [17]. All other measurements were log-transformed for subsequent analyses. Total leg length (tll) is the sum of the lengths of femur, tibiotarsus, and tarsometarsus. Total neck length (neck) is the sum of all vertebral lengths. The central vertebra is identified by dividing the cervical vertebral column into two parts. Refer to the material & methods section of the main manuscript for more details.

| Taxon | ID | | log_BM^1/3^ | log_tll | log_femur | log_tibiotarsus | log_tarsometatarsus | log_neck | log_central vertebra |
| --- | --- | --- | --- | --- | --- | --- | --- | --- | --- |
| *Amazona amazonica* | MNHN ZO | 2009-833 | -0.13874488 | -0.92705197 | -1.36713796 | -1.251192 | -1.71511829 | -1.17018181 | -2.1260984 |
| *Amazona autumnalis* | MNHN ZO | 2000-262 | -0.13282774 | -0.92223251 | -1.37099838 | -1.24169382 | -1.70487292 | -1.20502426 | -2.14935377 |
| *Amazona dufresniana* | MNHN ZO | 2004-198 | -0.08707315 | -0.82663888 | -1.27425167 | -1.1437546 | -1.61870438 | -1.08465294 | -2.04143612 |
| *Amazona vinacea* | MNHN ZO | 2000-250 | -0.13931221 | -0.89715484 | -1.34543845 | -1.21190237 | -1.69486368 | -1.14923127 | -2.08990945 |
| *Anas acuta* | MNHN ZO | 1986-35 | -0.02352703 | -0.82739707 | -1.38774609 | -1.16761884 | -1.39935376 | -0.81341091 | -1.88605665 |
| *Andigena nigrirostris* | MNHN ZO | 1986-38 | -0.1515643 | -0.7540927 | -1.29946934 | -1.11339649 | -1.31015859 | -0.97465317 | -1.93255716 |
| *Anhinga melanogaster* | MNHN ZO | 1883.543 | 0.05243521 | -0.72642005 | -1.24283181 | -1.06419055 | -1.35340025 | -0.46351731 | -1.75970042 |
| *Anodorhynchus hyacinthinus* | MNHN ZO | 1999-144 | 0.06483811 | -0.68742071 | -1.1490476 | -1.01908806 | -1.41184038 | -0.99636716 | -1.95781841 |
| *Anorrhinus galeritus* | MNHN ZO | 2000-302 | 0.02524313 | -0.65253337 | -1.15384852 | -1.00291957 | -1.27515091 | -0.84420865 | -1.8431481 |
| *Anseranas semipalmata* | MNHN ZO | 2004-151 | 0.14905268 | -0.50912892 | -1.09065796 | -0.86157098 | -1.0411973 | -0.60851796 | -1.74208155 |
| *Aprosmictus erythropterus* | MNHN ZO | 1997-925 | -0.26895847 | -1.00872961 | -1.48905305 | -1.32938311 | -1.72723041 | -1.21459898 | -2.17717836 |
| *Apteryx mantelli* | BMNH | 17-01-72-1 | 0.16610352 | -0.54060751 | -1.03971954 | -0.89469424 | -1.15926677 | -0.84034259 | -1.90274269 |
| *Apus apus* | MNHN ZO | 1997-995 | -0.46065063 | -1.27115943 | -1.74787545 | -1.59894427 | -1.97839728 | -1.44733178 | -2.45842076 |
| *Aquila chrysaetos* | MNHN ZO | 2013-362 | 0.18920481 | -0.45974563 | -0.88883834 | -0.88880472 | -1.0526153 | -0.83003183 | -1.78277934 |
| *Ara ararauna* | MNHN ZO | 1995-233 | 0.02487787 | -0.75631719 | -1.21211465 | -1.0835987 | -1.50293206 | -1.06308432 | -1.96940028 |
| *Ara macao* | MNHN ZO | 1995-262 | 0.0257893 | -0.7654321 | -1.21896306 | -1.09685573 | -1.50570623 | -1.02420069 | -1.97633608 |
| *Ara militaris* | MNHN ZO | 2011-357 | 0.00747612 | -0.78528858 | -1.24139086 | -1.11565785 | -1.52331326 | -1.05700041 | -2.01954211 |
| *Aramides cajanea* | MNHN ZO | 1880-1395 | -0.12977995 | -0.58986414 | -1.09974214 | -0.9515582 | -1.18151018 | -0.84218072 | -1.85636077 |
| *Ardea cinerea* | MNHN ZO | 1993-103 | 0.06311664 | -0.36468719 | -1.06419055 | -0.70289635 | -0.83159092 | -0.42604981 | -1.53003079 |
| *Baryphthengus ruficapillus* | MNHN ZO | 1997-920 | -0.27904567 | -0.92554928 | -1.46382047 | -1.28785559 | -1.48425858 | -1.20460667 | -2.19246497 |
| *Bombycilla garrulus* | MNHN ZO | 2010-115 | -0.40849435 | -1.12627262 | -1.64569944 | -1.47172622 | -1.73494621 | -1.43640027 | -2.29843202 |
| *Bubo bubo* | MNHN ZO | 2000-343 | 0.14418949 | -0.48359685 | -0.99744519 | -0.83129632 | -1.09506817 | -0.96237433 | -1.8951716 |
| *Burhinus oedicnemus* | MNHN ZO | 2009-801 | -0.12819202 | -0.66786464 | -1.30469348 | -1.04157947 | -1.12842706 | -1.10474325 | -2.07883395 |
| *Calonectris diomedea* | MNHN ZO | 2004-413 | -0.0634801 | -0.71586266 | -1.32873457 | -1.05963295 | -1.23440595 | -0.93386051 | -1.91865269 |
| *Calyptomena viridis* | MNHN ZO | 1997-886 | -0.41344405 | -1.0790944 | -1.55021315 | -1.39891827 | -1.81616096 | -1.32873457 | -2.25181197 |
| *Caprimulgus europaeus* | MNHN ZO | 1995-250 | -0.36837678 | -1.14593699 | -1.64149409 | -1.49322446 | -1.78225293 | -1.41805034 | -2.33724217 |
| *Caracara plancus* | MNHN ZO | 1979-107 | -0.01630181 | -0.55171117 | -1.14898639 | -0.94237193 | -1.01963297 | -0.96213544 | -1.9710223 |
| *Cariama cristata* | MNHN ZO | 1934-614 | 0.05869709 | -0.29682904 | -1.07644514 | -0.65091683 | -0.70421306 | -0.70031841 | -1.72078949 |
| *Casuarius casuarius* | MNHN ZO | 1947-72 | 0.58780933 | -0.02181948 | -0.61261017 | -0.41228904 | -0.49485002 | -0.42772102 | -1.57202729 |
| *Catharacta skua* | MNHN ZO | 2000-344 | 0.04870935 | -0.6474509 | -1.23143589 | -1.00256993 | -1.17334221 | -0.91108465 | -1.91115544 |
| *Charmosyna pulchella* | MNHN ZO | 1997-936 | -0.51005933 | -1.27197105 | -1.73660067 | -1.59756665 | -2.00612309 | -1.51769823 | -2.44733178 |
| *Chauna chavaria* | MNHN ZO | 1921-255 | 0.16610352 | -0.37860559 | -0.99714307 | -0.72955409 | -0.88223171 | -0.63859328 | -1.77624455 |
| *Ciconia ciconia* | MNHN ZO | 1997-940 | 0.17501494 | -0.24621018 | -0.99580764 | -0.58502665 | -0.68550077 | -0.52730205 | -1.59057413 |
| *Columba livia* | MNHN ZO | 1876-642 | -0.18954541 | -0.7503859 | -1.25235518 | -1.10507502 | -1.36421476 | -0.93835907 | -1.92118082 |
| *Coracopsis nigra* | MNHN ZO | 1883-507 | -0.19225138 | -0.94351441 | -1.43027505 | -1.25531594 | -1.67345933 | -1.16392919 | -2.1090204 |
| *Coragyps atratus* | MNHN ZO | 1923-13 | 0.05127162 | -0.54059243 | -1.10056255 | -0.87817762 | -1.11747546 | -0.82985568 | -1.86934465 |
| *Corvus corax* | MNHN ZO | 1997-949 | 0.03714352 | -0.60216424 | -1.1534155 | -0.95459878 | -1.16316971 | -0.97576069 | -1.9175737 |
| *Crypturellus cinereus* | MNHN ZO | 2004-187 | -0.12050358 | -0.69024212 | -1.20252471 | -1.08202212 | -1.23254728 | -0.94962024 | -2.05453142 |
| *Cyanoliseus patagonus* | MNHN ZO | 1996-26 | -0.1845394 | -0.905459 | -1.37365963 | -1.23121395 | -1.63264408 | -1.19962665 | -2.1260984 |
| *Cygnus olor* | MNHN ZO | MNHN1871-420 | 0.34447459 | -0.29551488 | -0.95726702 | -0.55268689 | -0.93572925 | -0.19486631 | -1.48691564 |
| *Dendrocopos major* | MNHN ZO | 2000-283 | -0.35857357 | -1.19702115 | -1.71624662 | -1.58419227 | -1.73849923 | -1.35822853 | -2.31875876 |
| *Dendrocopos medius* | MNHN ZO | 1997-526 | -0.36563667 | -1.05276239 | -1.58502665 | -1.42724454 | -1.59911678 | -1.43497907 | -2.33348202 |
| *Deroptyus accipitrinus* | MNHN ZO | 1997-433 | -0.20361131 | -0.95967462 | -1.42296801 | -1.28299559 | -1.70223949 | -1.22366209 | -2.17783192 |
| *Dromaius novaehollandiae* | MNHN ZO | 1897-89 | 0.58655595 | -0.03007181 | -0.6838199 | -0.42481216 | -0.45593196 | -0.25824192 | -1.48386142 |
| *Dryocopus martius* | MNHN ZO | 2013-344 | -0.18847703 | -0.88779731 | -1.37923951 | -1.28449806 | -1.44635966 | -1.09178315 | -2.02918839 |
| *Eudyptes chrysocome* | MNHN ZO | 1997-112 | 0.15413267 | -0.73776255 | -1.19395576 | -1.02951365 | -1.59345982 | -0.9516359 | -1.95116991 |
| *Eurypyga helias* | MNHN ZO | 1869-396 | -0.24194738 | -0.78036309 | -1.44105155 | -1.12101888 | -1.26825012 | -0.88505558 | -1.89448982 |
| *Falculea palliata* | MNHN ZO | 1883-531 | -0.31628249 | -0.96509093 | -1.5182708 | -1.33059013 | -1.50390101 | -1.19708951 | -2.15552282 |
| *Fregata ariel* | MNHN ZO | 1997-178 | -0.04164625 | -0.92800759 | -1.37809704 | -1.23210238 | -1.75547549 | -0.9056337 | -1.88472241 |
| *Gallirallus australis* | MNHN ZO | 1870-182 | -0.01638285 | -0.61459037 | -1.11970101 | -0.97138809 | -1.22061999 | -0.84618514 | -1.86486735 |
| *Gallus gallus* | MNHN ZO | 1893-232 | 0.0085718 | -0.44070397 | -0.98733147 | -0.82364824 | -0.96082392 | -0.68954036 | -1.72307887 |
| *Gavia arctica* | MNHN ZO | 1992-9 | 0.12368929 | -0.60537324 | -1.36071277 | -0.90423804 | -1.09772508 | -0.9516359 | -1.65915945 |
| *Geronticus eremita* | MNHN ZO | 1997-750 | 0.05408854 | -0.58377568 | -1.16647042 | -0.92223251 | -1.13679614 | -0.81423127 | -1.86486735 |
| *Glareola pratincola* | MNHN ZO | 2009-803 | 0.01046949 | -1.05222329 | -1.6367642 | -1.4205594 | -1.55877633 | -1.42227848 | -2.39040559 |
| *Grus grus* | MNHN ZO | 1997-102 | 0.24939601 | -0.16857818 | -0.88840148 | -0.53209605 | -0.59294919 | -0.40904677 | -1.47586362 |
| *Gypaetus barbatus* | MNHN ZO | 1992-2 | 0.254476 | -0.45069014 | -0.95506845 | -0.81559252 | -1.04359143 | -0.79056542 | -1.79997073 |
| *Gyps fulvus* | MNHN ZO | 1996-43 | 0.254476 | -0.35310638 | -0.85328296 | -0.7212464 | -0.94573176 | -0.54890469 | -1.62451929 |
| *Gyps rueppellii* | MNHN ZO | 1997-948 | 0.29920903 | -0.33136253 | -0.81835646 | -0.69897 | -0.94180181 | -0.59593639 | -1.65915945 |
| *Haematopus ostralegus* | MNHN ZO | 1994-36 | -0.04785774 | -0.74586297 | -1.33077613 | -1.09533854 | -1.27942728 | -1.0216825 | -2.01457353 |
| *Himantopus himantopus* | MNHN ZO | 1986-41 | -0.24388536 | -0.53766191 | -1.46750041 | -0.88163606 | -0.90465627 | -0.99473367 | -1.99182582 |
| *Larus glaucescens* | MNHN ZO | 1993-346 | -0.36745764 | -0.57298479 | -1.15558496 | -0.910837 | -1.12708656 | -0.7654068 | -1.77391588 |
| *Lorius garrulus* | MNHN ZO | 1996-137 | -0.22252051 | -0.96050657 | -1.42527442 | -1.29056043 | -1.68319125 | -1.18170811 | -2.11182051 |
| *Lybius dubius* | MNHN ZO | 1997-879 | -0.3469862 | -1.01781925 | -1.58703573 | -1.36261035 | -1.57332611 | -1.3080349 | -2.29499204 |
| *Megaceryle torquata* | MNHN ZO | 2004-196 | -0.07601508 | -1.00948356 | -1.45419824 | -1.32413005 | -1.81559252 | -1.09832377 | -2.09745322 |
| *Megapodius cumingii* | MNHN ZO | 1884-379 | 0.17217846 | -0.63895246 | -1.17082493 | -1.00992767 | -1.19097928 | -0.92281385 | -1.96417018 |
| *Merops apiaster* | MNHN ZO | 1997-1080 | -0.40489006 | -1.24526953 | -1.70774393 | -1.5965363 | -1.92335956 | -1.41578189 | -2.35753548 |
| *Mitu tuberosum* | MNHN ZO | 1997-333 | 0.1955291 | -0.40021515 | -0.94861592 | -0.76853041 | -0.93949102 | -0.6134832 | -1.63732907 |
| *Morus bassanus* | MNHN ZO | 1997-191 | 0.22556454 | -0.63165732 | -1.1382267 | -0.99186844 | -1.22988471 | -0.62085771 | -1.70996539 |
| *Neophron percnopterus* | MNHN ZO | 1995-229 | 0.01379756 | -0.5754777 | -1.13235577 | -0.94838448 | -1.10001544 | -0.92679504 | -1.94846161 |
| *Numida meleagris* | MNHN ZO | 1885-332 | 0.0461009 | -0.55462935 | -1.06048075 | -0.93352499 | -1.12314735 | -0.78175342 | -1.82419837 |
| *Opisthocomus hoazin* | MNHN ZO | 1997.802 | -0.03230334 | -0.68411358 | -1.19111413 | -1.04958646 | -1.27286558 | -0.93726764 | -1.9905491 |
| *Oriolus oriolus* | MNHN ZO | 1993-131 | -0.38088917 | -1.08291481 | -1.59277911 | -1.44454278 | -1.67468963 | -1.19708951 | -2.3080349 |
| *Otis tarda* | MNHN ZO | 1875-784 | 0.35851565 | -0.30365235 | -0.89966392 | -0.67736729 | -0.79371396 | -0.61469766 | -1.61493022 |
| *Pandion haliaetus* | MNHN ZO | 1996-49 | 0.06803999 | -0.6067232 | -1.11605481 | -0.92357803 | -1.28785559 | -0.93824603 | -1.95900231 |
| *Pelecanus crispus* | MNHN ZO | 1995-278 | 0.35356595 | -0.36849535 | -0.89506926 | -0.74593555 | -0.9163899 | -0.21442105 | -1.3471737 |
| *Phaethon aethereus* | MNHN ZO | 1997-198 | -0.05163399 | -0.91861668 | -1.4225082 | -1.27067319 | -1.53476591 | -1.03498655 | -2.01278077 |
| *Phalacrocorax verrucosus* | MNHN ZO | 1979-191 | 0.11414089 | -0.63065425 | -1.19654288 | -0.95565627 | -1.2238802 | -0.6799992 | -1.7883456 |
| *Phoenicopterus ruber* | MNHN ZO | 1997-205 | 0.16378723 | -0.15765916 | -1.02747318 | -0.51173139 | -0.53180041 | -0.35714983 | -1.3819519 |
| *Picus viridis* | MNHN ZO | 1997-1126 | -0.23739942 | -0.97155076 | -1.49241396 | -1.35251923 | -1.52028077 | -1.18170811 | -2.15181088 |
| *Platycercus elegans* | MNHN ZO | 1996-134 | -0.29042591 | -1.02209357 | -1.50514998 | -1.35941919 | -1.69723629 | -1.33818731 | -2.20342567 |
| *Platycercus eximius* | MNHN ZO | 1997-923 | -0.32627023 | -1.0437355 | -1.54272381 | -1.36511986 | -1.73002032 | -1.33818731 | -2.25570702 |
| *Pluvialis apricaria* | MNHN ZO | 1997-1171 | -0.21529719 | -0.85118149 | -1.43640027 | -1.20059052 | -1.38457605 | -1.3283644 | -2.2636035 |
| *Podiceps cristatus* | MNHN ZO | 1997-118 | 0.00609477 | -0.68875488 | -1.36865771 | -1.00300702 | -1.20300961 | -0.65856548 | -1.80715389 |
| *Poicephalus gulielmi* | MNHN ZO | A9223 | -0.185579 | -0.96842997 | -1.42308304 | -1.2964507 | -1.71534372 | -1.24146658 | -2.17783192 |
| *Poicephalus robustus* | MNHN ZO | 2004-197 | -0.16815189 | -0.9265016 | -1.37396775 | -1.25696085 | -1.68131073 | -1.23210238 | -2.15428198 |
| *Poicephalus senegalus* | MNHN ZO | 1881-147 | -0.28410789 | -1.03574037 | -1.49689056 | -1.36181036 | -1.77520804 | -1.32532253 | -2.26520017 |
| *Psittacula krameri* | MNHN ZO | 1997-439 | -0.30815101 | -1.02452224 | -1.48638293 | -1.34949821 | -1.76548272 | -1.27753061 | -2.24488773 |
| *Psittacus erithacus* | MNHN ZO | 1997-445 | -0.11688838 | -0.8645811 | -1.31921139 | -1.18943147 | -1.61996975 | -1.14490505 | -2.1001795 |
| *Pterocles quadricinctus* | MNHN ZO | 1878-598 | -0.2482425 | -0.94036086 | -1.4015378 | -1.3165927 | -1.57170319 | -1.20259395 | -2.21968269 |
| *Rhea americana* | MNHN ZO | 1993-6 | 0.45072751 | -0.12557617 | -0.73189027 | -0.53342893 | -0.56751174 | -0.38411305 | -1.45419824 |
| *Rhynochetos jubatus* | MNHN ZO | 1886-128 | -0.0152525 | -0.54637593 | -1.16800232 | -0.92241409 | -1.01448371 | -0.90059563 | -1.90868484 |
| *Sagittarius serpentarius* | MNHN ZO | 1892-965 | 0.17217846 | -0.171295 | -0.94703687 | -0.5529971 | -0.55098468 | -0.68552182 | -1.73400363 |
| *Sarcoramphus papa* | MNHN ZO | 1995-03 | 0.17609126 | -0.4490871 | -1.00397017 | -0.79398412 | -1.01877051 | -0.76603986 | -1.81191563 |
| *Scolopax rusticola* | MNHN ZO | 1997-1107 | -0.18662613 | -0.83283041 | -1.33639329 | -1.20210952 | -1.41941712 | -1.14849705 | -2.1408617 |
| *Scopus umbretta* | MNHN ZO | 1896-300 | -0.12472443 | -0.70679218 | -1.34601609 | -1.04522701 | -1.21296482 | -0.97802555 | -1.90101036 |
| *Struthio camelus* | MNHN ZO | 1908-160 | 0.70240332 | 0.1271048 | -0.50031292 | -0.2588484 | -0.32513886 | -0.03589129 | -1.23239893 |
| *Tauraco corythaix* | MNHN ZO | 1996-129 | -0.2205145 | -0.79347095 | -1.28332902 | -1.15027356 | -1.41953122 | -1.10773839 | -2.07468791 |
| *Thalassarche melanophrys* | MNHN ZO | 1889-34 | 0.20068666 | -0.49724523 | -1.13047494 | -0.81852801 | -1.0347042 | -0.77780395 | -1.76396685 |
| *Tyto alba* | MNHN ZO | 1997-494 | -0.16449832 | -0.72126926 | -1.30697693 | -1.08698132 | -1.23047498 | -1.26416167 | -2.23433145 |
| *Upupa epops* | MNHN ZO | 1993-123 | -0.39023208 | -1.12436006 | -1.65995268 | -1.47820835 | -1.69962194 | -1.23395914 | -2.22914799 |
| *Uria lomvia* | MNHN ZO | 1993-347 | -0.0081894 | -0.75654027 | -1.30155146 | -1.06560256 | -1.40616034 | -0.98750022 | -1.98046832 |
| *Vanellus vanellus* | MNHN ZO | 1997-1150 | -0.21338817 | -0.81987413 | -1.42770939 | -1.17757038 | -1.32230182 | -1.20551195 | -2.19517932 |
| *Vultur gryphus* | MNHN ZO | 1973-202 | 0.35038417 | -0.31461659 | -0.83238733 | -0.66294027 | -0.92009553 | -0.54947377 | -1.59345982 |

**Supplementary material references**

1. Taylor M.P., Wedel M.J. 2013 The effect of intervertebral cartilage on neutral posture and range of motion in the necks of sauropod dinosaurs. *PLoS One* **8**(10), e78214. (doi:10.1371/journal.pone.0078214).

2. Wilman H., Belmaker J., Simpson J., de la Rosa C., Rivadeneira M.M., Jetz W. 2014 EltonTraits 1.0: Species-level foraging attributes of the world's birds and mammals. *Ecology* **95**(7), 2027. (doi:10.1890/13-1917.1).

3. De Graaf R.M., Tilgham N.G., Anderson S.H. 1985 Foraging guilds of North American birds. *Environmental management* **9**(6), 493-536.
